# Fronto-parietal connectivity changes following noxious stimulation during anesthesia

**Published:** 2014-09-25

**Authors:** B Pavel, A Daneasa, AE Rosca, A Calin, D Zahiu, A Panaitescu, AM Zagrean, L Zagrean

**Affiliations:** "Carol Davila" University of Medicine and Pharmacy, Bucharest, Romania

**Keywords:** cortical connectivity, anesthesia, noxious stimulation

## Abstract

Abstract

Rationale: The aim of our study was to assess the changes in the fronto-parietal connectivity estimated by the cross approximate entropy (XAppEn) during noxious stimulation while under chloral hydrate anaesthesia, in rats.

Method: A group of 11 Wistar rats chronically implanted with Ni-Cr electrodes, which were placed on the dura mater of the right hemisphere (over the olfactory cortex, the frontal and the parietal lobes), were used in the present study. Noxious stimuli of a mechanical and thermal nature were applied on the left hindpaw during chloral hydrate anesthesia. The anesthetic depth was estimated through median frequency computation, which in that instance was of 2-3 Hz. Fronto-parietal functional cortical connectivity was assessed by using XAppEn.

Results: After data processing and analysis we observed an increase of fronto-parietal functional connectivity during mechanical and thermal noxious stimulation. In addition, MEF increased both in frontal and parietal areas during the mechanical and thermal stimulation compared to baseline.

Conclusion: Mechanical and thermal stimulation induces an increase in the fronto-parietal connectivity during chloral hydrate anesthesia in rats.

## Introduction

Cortical and subcortical connectivity decreases in parallel with anesthetic depth [**1-3**]. Fronto-parietal connectivity, which is considered the most important substratum of consciousness, is impaired during anesthesia, especially the feedback within the network [**4,5**]. The two principal methods used to assess cortical connectivity in humans and animals are fMRI and EEG recordings, both having similar accuracy [**6**]. In this study, we opted for Cross Approximate entropy (XAppEn) analysis from EEG data as the functional connectivity parameter. Cross Approximate entropy is a newly introduced parameter used to quantify the correlation within different biological signals [**7-9**]. A previous EEG study reported a decrease of the cortical connectivity assessed by the XAppEn during anesthesia, the decrease corresponding to anesthesia depth [**10**]. There is no information about how noxious stimulation affects this connectivity. In a previous paper/study (in press), we reported that fronto-parietal connectivity, assessed by using the correlation coefficient, did not change during noxious stimulation compared to baseline, in spite of cortical activation. In this study, we intended to evaluate the changes in fronto-parietal connectivity following mechanical and thermal noxious stimulation, during chloral hydrate anesthesia in rats. As far as we know, such a study has not been conducted yet.

## Material and methods

Animals

 A group (n=11) of Wistar rats with weights ranging from 250 to 300 grams were used. They were kept in a room with constant temperature (23° Celsius) and 12-h light-dark cycle (lights on at 07:00 h), with free access to food and water. Rats were chronically implanted, under chloral hydrate (Sigma-Aldrich Gmbh, Munich, Germany) anesthesia (400 mg/kg, injected intraperitoneal), using Nickel-Chrome (Ni80Cr20, Ø 0.15 mm, Goodfellow, UK) electrodes which were placed directly on the dura mater, through burr holes obtained by trephination, and were finally fixed with cement. Three electrodes were implanted, one reference electrode was placed over the olfactory cortex (7 mm anterior to bregma and 1 mm lateral to the frontal suture) and two active electrodes were placed over the right hemisphere, one on the frontal lobe (3 mm anterior to bregma and 3 mm lateral to the frontal suture), corresponding to the motor cortex, and the other on the parietal lobe (3 mm posterior to bregma and 3 mm lateral to the sagittal suture), corresponding to the somatosensory areas of the left hindpaw [**11**]. The rats underwent experimentation after a minimum period of 7 days after the intervention. This study has been approved by the Ethics Committee of "Carol Davila" University of Medicine and Pharmacy Bucharest.

 Anesthesia and stimulation procedure

 Anesthesia was induced and maintained with chloral hydrate (300 mg/kg BW administrated intra peritoneal). After a steady state of 20 minutes from the induction and after the desired anesthetic depth was reached, which was estimated by calculating the MEF of the frontal lobe EEG track, in this case the target being 2-3Hz, we started the recording. The mechanical stimulus consisted in placing the skin from the posterior left hindpaw between the jaws of a Pean type forceps, and closing it until the first ratchet locked, for 10 seconds. The thermal stimulus consisted in placing a resistor warmed up to 57º Celsius on the left hindpaw for 10 seconds. A 5 minute interval passed between the two stimuli. Recording continued for another 2 minutes after the thermal stimulus ended. During the experiment, the rats’ temperature was maintained at 37º - 38º Celsius by using a heating pad.

 Data acquisition and signal processing

 The electrocorticogram (ECoG) signal acquisition was made by using BIOPAC MP-150 system (Biopac Systems, CA, USA) at a sample rate of 1000 Hz, a gain of 1000, with filters set at 0.5 Hz for high-pass and 30 Hz for low-pass. Recordings were saved in .mat format and imported into Matlab. XAppEn between frontal and parietal area signals was computed by using Kreuzer’s script for Matlab [**10**]. The analysed data consisted of a 2 seconds long selection from a 1 minute baseline signal, cropped from the middle of the signal, and 2 seconds long selections from the 10 second long signals corresponding to the noxious stimulation, one for each mechanical and thermal, again cropped from the middle of the signal. We compared the mean value of the XAppEn during baseline with those during mechanical and thermal stimulus application.

 Statistical analysis

 Data was represented as mean value ± standard deviation (SD). Statistical analysis was performed by using the t-test. The software used was SPSS Statistics 17. P values <0.05 were considered statistically significant.

 The material and method described above were also described in one of our previous studies [in press].

## Results

**Fig. 1:**
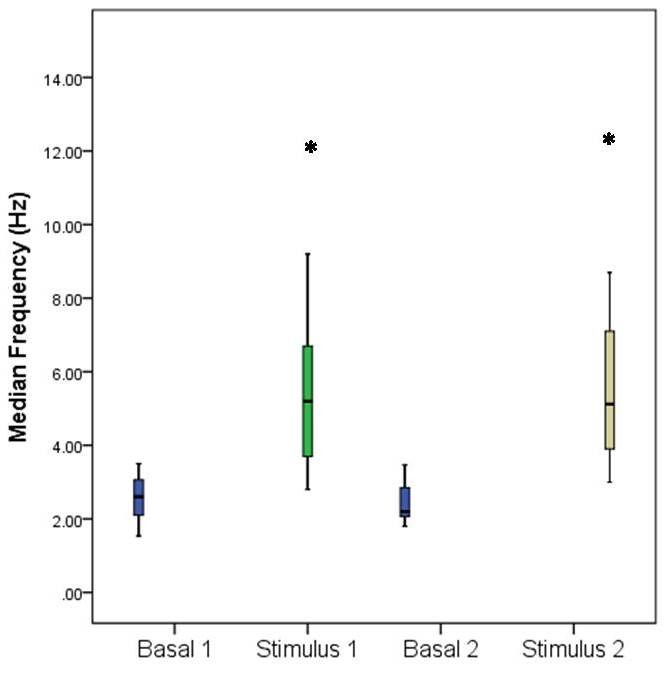
Median frequency (MEF) variation in the frontal lobe following noxious stimulation. Basal 1 represents the value of MEF during baseline before mechanical stimulation, Stimulus 1 represents the value of MEF during mechanical stimulation, Basal 2 represents the value of MEF during baseline before thermal stimulation, Stimulus 2 represents the value of MEF during thermal stimulation. Error bars represent S.D. Stars indicate statistical significance (p<0.05).

**Fig. 2:**
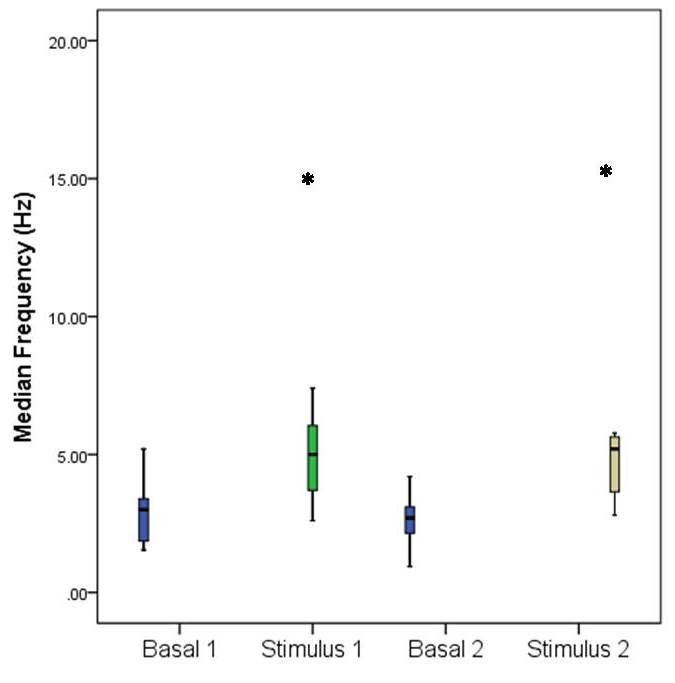
Median frequency (MEF) variation in the parietal lobe following noxious stimulation. Basal 1 represents the value of MEF during baseline before mechanical stimulation, Stimulus 1 represents the value of MEF during mechanical stimulation, Basal 2 represents the value of MEF during baseline before thermal stimulation, Stimulus 2 represents the value of MEF during thermal stimulation. Error bars represent S.D. Stars indicate statistical significance (p<0.05).

 Data processing revealed an increase of MEF during mechanical and thermal noxious stimulation, compared to baseline, MEF increasing in both frontal and parietal area as shown in **Fig. 1** and **Fig. 2**. 

**Fig. 3:**
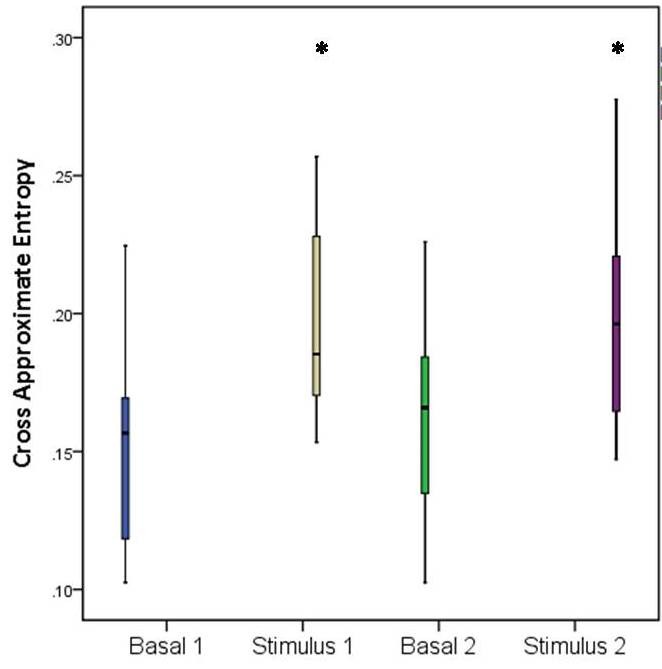
Cross Approximate Entropy (XAppEn) variation during mechanical and noxious stimulation. Basal 1 represents XAppEn between frontal and parietal cortex during baseline, Stimulus 1 represent XAppEn between frontal and parietal cortex during mechanical stimulation, Basal 2 represents XAppEn between frontal and parietal cortex during the baseline before thermal stimulation, Stimulus 2 represents XAppEn between frontal and parietal cortex during thermal noxious stimulation. Error bars represent S.D. Stars indicate statistical significance (p<0.05).

 This MEF increase is statistically significant (p<0.05). In the frontal area MEF increased from a baseline value of 2.76 ± 1 Hz to 5.98 ± 3.38 Hz during mechanical stimulation (p=0.005) and from 2.47 ± 0.64 Hz to 5.93 ± 2.9 Hz during thermal stimulation (p=0.001). The same trend can be observed in the parietal area, where MEF varied from a baseline value of 2.95 ± 1.14 Hz to 5.64 ± 3.53 Hz during mechanical stimulation (p=0.016), and from 2.71 ± 0.92 to 5.30 ± 2.16 during thermal stimulation (p=0.001). XAppEn computing showed an increase during noxious stimulation from a baseline value of 0.151 ± 0.037 to 0.198 ± 0.035 (p=0.0034) during mechanical stimulation, and from a baseline value of 0.16 ± 0.039 to 0.199 ± 0.043 (p=0.018) during thermal stimulation (**Fig. 3**).

## Discussion

 Following data processing and analysis, an increase in ECoG activity in the parietal and frontal lobes was observed. Similarly, median frequency increases in the parietal and frontal lobe compared to baseline, independent of the nature, mechanical or thermal, of the noxious stimulus. Mechanical stimulation inconsistently generates MEF changes during halothane anesthesia [**12,13**]. This inconsistency depends on the type of stimulus that is used, MEF increasing only during oscillating clamp application. We obtained an increase in MEF during stationary clamp application which has not been reported by other studies. This could be due to the fact that the stimulus time used in our protocol was of 10 seconds, twice as long as the 5 second stimulation used in the other studies. Another pertinent difference is the type of anesthetic that we used, namely chloral hydrate [in press]. It has been proved that thermal stimulation generates EEG changes during anesthesia. Under halothane anesthesia in rats, tail thermal stimulation induced EEG activation in some cortical areas (rights somatosensory and vertices areas) [**12**]. A recent human study on thermal noxious stimulation effects on EEG under anesthesia, using bispectral (BIS) analysis score, found that the thermal stimulation at 40/100 and 70/100 intensity produced BIS score increases, but without a significant difference between these two stimuli [**14**]. In our study, the MEF increased in the frontal as well as in the parietal area, similar to the response observed during mechanical stimulation. These findings are in accordance with previous reports [**12**]. The similarity of the MEF increase during mechanical and thermal stimulation could be explained by the supramaximal nature of these stimuli [**12**].

 Cortical and subcortical connectivity is known to decrease during anesthesia [**15**]. The drop in connectivity between the frontal and parietal areas is considered the most relevant factor in determining the loss of consciousness [**3-5**]. In the current study, the assessment of the functional connectivity between frontal and parietal cortical areas was performed by using XAppEn algorithm [**10**]. The difference between the XAppEn computing result during noxious stimulation and that during baseline was statistically significant. The reason we obtained a connectivity increase during noxious stimulation is in all likelihood a decrease in the depth of the anesthesia. MEF increase suggested a lightening of anesthesia, a situation in which functional connectivity is known to increase; therefore, functional connectivity between frontal and parietal areas increases because noxious stimuli lightened the anesthesia. This lightened anesthesia could be generated by the supramaximal noxious stimulation and the inadequate anesthetic depth. Mechanical (mechanical clamp) and thermal (57° Celsius) noxious stimuli are considered to be supramaximal according to other, previous report [**12**]. In comparison with our previous experiment [in press] in which the functional connectivity was analysed by using a correlation coefficient which did not significantly change during stimulation compared to baseline, our present study found an increase in functional connectivity during the mechanical stimulation. In both our studies, present and past, mechanical stimulation induced an increase in MEF and caused a lightening of anesthesia. Therefore, we interpreted these different results to be due to a higher sensitivity of XAppEn for the detection of functional connectivity change, compared to the correlation coefficient.

## Conclusion 

 Mechanical and thermal noxious stimulation generated an increase in MEF both in the frontal and parietal areas, when applied during chloral hydrate anesthesia. Analysis of functional cortical connectivity by using XAppEn showed an increase in connectivity during stimuli application, both thermal and mechanical. XAppEn is a better parameter than the correlation coefficient for functional connectivity assessment during mechanical noxious stimulation.
